# Emergency Ultrasound: Is It Time for Artificial Intelligence?

**DOI:** 10.3390/jcm11133823

**Published:** 2022-07-01

**Authors:** Andrea Boccatonda

**Affiliations:** Internal Medicine, Bentivoglio Hospital, AUSL Bologna, 40010 Bologna, Italy; andrea.boccatonda@ausl.bo.it; Tel.: +39-051664-4111

Ultrasound is a fundamental and indispensable diagnostic method in the field of emergency medicine. A large amount of data collected in recent years has shown how ultrasound evaluation represents the fifth pillar of physical examination and how the ultrasound probe may become the stethoscope of the new millennium. This type of ultrasound is based on the characteristics of simplicity, speed, and reproducibility.

The concept of point-of-care ultrasound (POCUS) is based on the premise of completing physical examination with an ultrasound evaluation in order to answer simple but clinically relevant questions. The ultrasound is performed personally by the clinician who manages the patient, and can be carried out directly at the patient’s bed (bedside). Furthermore, this type of evaluation can be repeated as many times as needed. One of the most important features of this method is its ability to enable multi-organ and integrated evaluation by the clinician. For example, when a physician faced with a patient with hypoperfusion of unknown origin, the evaluation of the vena cava, the heart, the thoracic and abdominal cavity, and the venous system of the limbs allows us to reach a presumed etiological diagnosis in most cases. Furthermore, ultrasound monitoring is very relevant for therapeutic management, especially to identify the right fluid therapy. Finally, ultrasound is a very useful support tool for several invasive procedures, thus increasing success rates and significantly reducing complications.

This type of ultrasound requires specific training based on theory and practical tests. One of the limits, probably the greatest, which has always been related to ultrasound is that of interpersonal variability, meaning that it depends on the skills of the examining physician and the patient’s condition.

In the latter, computer-based artificial intelligence (AI) has become a leading research focus within the scientific community. It is used to carry out a series of assessments and measurements of aspects that are most affected by inter-individual variability. AI is a branch of computer science that includes machine learning (ML), deep learning (DL), and convolutional neural networks (CNNs). AI can employ devices to imitate the human cognitive process, such as learning, applying, and solving complex problems. An example is this of the evaluation of some specific measurements in the field of echocardiography, or its use in the field of lung ultrasound for the quantification of vertical artifacts in suspected interstitial syndrome [1].

Indeed, some data have shown that AI is an excellent tool for providing quantitative assessment automatically, thus reporting more accurate and reproducible results.

AI-based technology in the field of US imaging has been demonstrated to be successful for the noninvasive detection of cancer in patients, evaluating the degree of malignancy, or predicting prognosis [2]. 

In a recent work by Zhang et al. [3], researchers employed the DL model to fully automate the processing of echocardiography, including disease detection, image segmentation, and structure and function quantification. 

Moreover, the DL-based assessment of vascular US images has been applied to the measurement and classification of carotid artery intima-media thickness, the classification of vascular plaque components, the detection of the vascular lumen, and the segmentation of vascular images [4,5]. 

In a recent work on lung ultrasound, seven novice learners (NLs) with limited to no prior POCUS experience completed examinations on 32 patients presenting to a pediatric ED with a cardiopulmonary chief complaint [6]. The sensitivity, specificity, and accuracy of NL AI-augmented interpretation were 66.7% (confidence interval (CI) 9.4–99.1%), 96.5% (CI 82.2–99.9%), and 93.7% (CI 79.1–99.2%) [6]. The interrater reliability between expert sonographers was high, with a kappa coefficient of 0.8 [6]. Therefore, the use of AI-augmented lung US for diagnosing pneumonia has the potential to increase accuracy and efficiency.

Another recent work tested an ultrasound lung imaging diagnosis model based on an artificial intelligence algorithm (DRN) in neonatal respiratory distress syndrome (NRDS) [7]. In children with NRDS, the positive rate of abnormal pleural line, the disappearance of A line, the appearance of B line, and alveolar interstitial syndrome test in the results of lung ultrasound examination in children with NRDS were all 100% [7]. The diagnostic model of this study predicted that the AUC areas of grades 1–2, grades 2–3, and grades 3–4 NRDS were 0.962, 0.881, and 0.902, respectively [7]. 

Therefore, the use of AI can improve the diagnostic performance of the operator, even when they have minimal experience and suboptimal training. While this technique represents a relevant advantage for the patient and for global health management, on the other hand it raises practical and concept concerns—is it possible to create a new type of diagnostic tool supported mainly by AI systems that can be used by support operators (including non-health professionals) with minimal training?

This ultrasound technology could be successful in complex situations from the point of view of geographical location and for operators characterized by limited resources and time, but is it possible to leave most of the diagnostic reasoning to the machine on a normal condition?

Some data have demonstrated the excellent performance of AI in detecting pathological signs and/or specific data, which are often affected by the operator’s experience and by inter-individual variability (e.g., EF quantification, the counting of B lines, or IMT determination). If these data clearly reinforce the benefits of the employment of AI in these fields, what is the relationship towards the diagnosis of complex multiorgan diseases?

In emergency setting more than in others, it is evident how an integrated multi-organ ultrasound represents a valuable tool in the face of a complex patient who reports a disease history involving a series of multiple signs and symptoms (e.g., the diagnosis of septic shock in the course of acute cholecystitis). Currently, complex and multi-organ clinical reasoning is not part of AI, and the final diagnosis is always made by the clinician, who integrates all the clinical, laboratory, and imaging information. A combination of the use of AI and human clinical reasoning is certainly desirable; indeed, the use of AI can provide more accurate and comparable data (e.g., ejection fraction or the count of B lines) compared to traditional evaluation, but these data must be included within an integrated clinical–ultrasound evaluation that only the physician can carry out.

One of the most relevant concerns about AI concerns image collecting. Indeed, the data contributing to the most central and critical component of AI learning systems, and thus both the quantity and quality of information in a database, will directly affect the performance of the AI-related technologies. Therefore, standards for the collecting and processing of ultrasound images should be created to improve quality of databases, especially across different institutions. 

All ultrasound devices, including the latest generation of pocket-sized ones, are equipped with an online connection system. This connection firstly provides users the possibility to send and store images and videos. These images can be evaluated from a distance and this opens up two possible scenarios: the first is that of telemedicine or teleultrasound—that is, the possibility of requesting remote evaluation by an external operator who could guide the operator in the field in how to carry out the examination; the second is that of distance learning, which became highly relevant during the COVID-19 pandemic, where the large-scale dissemination of lung ultrasound techniques was conducted online.

In the near future, AI-model technology able to merge 2D, 3D, SWE, and CEUS images called ultrasonic radiomics can provide the high-throughput medical features of detected diseases.

In conclusion, AI can represent a new tool for improving ultrasound performance, provided that the data obtained are processed by a clinician and integrated with other aspects of diagnostic evaluation, especially in the intensive care setting. Therefore, the use of AI in ultrasound does not represent the victory of machine over man, but rather a supporting tool for the enhancement of clinical reasoning.
